# Reply to the Letter to the Editor: Current use of percutaneous ablation in renal tumors—an analysis of the registry of the German Society for Interventional Radiology and Minimally Invasive Therapy

**DOI:** 10.1007/s00330-025-12163-2

**Published:** 2025-11-14

**Authors:** Benedikt M. Schaarschmidt, Claudia Kesch, Jonathan Nadjiri

**Affiliations:** 1 Institute of Diagnostic and Interventional Radiology and Neuroradiology, University Hospital Essen, Essen, Germany; 2 Department of Urology, University Hospital Essen, Essen, Germany; 3 Department of Interventional Radiology, Klinikum rechts der Isar, Technische Universität München, München, Germany

We thank Dr. Lou and Dr. Hu for their insightful letter on our study and appreciate the opportunity to respond to their remarks [1]. As acknowledged in the manuscript, our study does have certain limitations. We therefore welcome the chance to further clarify the study design and its practical constraints.

First, the study is based on the prospectively managed registry of the German Society for Interventional Radiology and Minimally Invasive Therapy (DeGIR), a database where information on various interventions can be submitted using an online form. At the moment, participation is restricted to sites in Austria, Germany, and Switzerland, but an expansion of the registry to other countries is currently underway. The advantages and disadvantages of studies based on such large-scale registries have been discussed very insightfully by Michael Vouche in his commentary on a prior study focusing on the thermal ablation of liver tumors [2, 3] and by Timo Auer and Thomas Kröncke in their commentary on our study [1, 4]. While voluntary large-scale registries provide the possibility to amass vast quantities of data by the low hurdles for participating centers, the value of this approach is limited by the lack of a central audit instance controlling and validating all inputs. Furthermore, we observed a marked increase in cryoablations in 2023, which might influence success rates due to a lack of experience by individual centers. Thus, we think that our analysis shall be primarily used for hypothesis generation or the validation of initial findings from pilot studies in a larger cohort. Therefore, we refrained from using mathematical model calculations, although this might be an interesting option in the future when more data has been added to the registry.

Second, Dr. Lou and Dr. Hu regret the lack of more robust endpoints (such as local recurrence rates, progression-free survival, or overall survival), technical details on the ablation procedures, and histopathological RCC subtypes. We totally agree that more detailed information on technical as well as histopathological details would improve the registry, but conclude that publication of large data sets is required at this time point to add adequately to the literature. Furthermore, a compact set of parameters within the registry allows for a vast participation with more than 210,000 entries in the registry per year, and therefore, big data studies as referenced. Here, the addition of new data points could cause compatibility issues with data collected in prior years. Still, augmentation and further development are discussed every year by the DeGIR to ensure a balance between usability of the registry, data coherence, and scientific needs.

Even though the number of interventions in the study is unparalleled, we agree with Dr. Lou and Dr. Hu that the value of such registries could be increased by outcome data. Here, a close collaboration with cancer registries could further increase the value of such registries. Until then, we consider this work an important milestone to highlight the clinical role of the interventional radiologists and the high procedural quality of percutaneous ablation in renal tumors. Further questions raised by studies based on large-scale registries should be included in prospective, clinical studies for further clarification.
